# Fatigue and sleep quality in different trimesters of pregnancy

**DOI:** 10.5935/1984-0063.20200091

**Published:** 2021

**Authors:** Fatemeh Effati-Daryani, Sakineh Mohammad-Alizadeh-Charandabi, Azam Mohammadi, Somayeh Zarei, Mojgan Mirghafourvand

**Affiliations:** 1 Reproductive Health Research Center, Faculty of Nursing and Midwifery, Urmia University of Medical Sciences, Department of Midwifery - Urmia - West Azarbaijan - Iran.; 2 Faculty of Nursing and Midwifery, Tabriz University of Medical Sciences, Department of Midwifery - Tabriz - East Azerbaijan - Iran.; 3 Ph.D. Student in Reproductive Health, Nursing and Midwifery Care Research Center, Faculty of Nursing and Midwifery, Tehran University of Medical Sciences, Department of Midwifery and Reproductive Health - Tehran - Tehran - Iran.; 4 Shohada Hospital, Qom University of Medical Sciences, Department of Midwifery - Qom - Qom - Iran.; 5 Social Determinants of Health Research Center, Tabriz University of Medical Sciences, Tabriz, East Azerbaijan, Iran.

**Keywords:** Fatigue, Sleep, Prevalence, Pregnancy

## Abstract

**Objectives:**

Fatigue is one of the most common and persistent complaints of pregnant women. Increased severity of fatigue during pregnancy can increase the risk of preterm labor, prolonged labor, cesarean section, and postpartum depression. Therefore, this study aimed to determine the fatigue status in different trimesters of pregnancy and its relationship with sleep quality in pregnant women.

**Material and Methods:**

This cross-sectional study was performed by two-stage cluster sampling on pregnant women referred to Tabriz health centers. Data were collected using socio-demographic questionnaire, multidimensional assessment of fatigue and Pittsburgh sleep quality index.

**Results:**

Among the 605 pregnant women studied, 94.2% of women suffered from fatigue during pregnancy. The mean (standard deviation) of fatigue score in the first, second and third trimesters of pregnancy was 23.53 (8.05), 25.78 (6.56) and 26.46 (6.72), respectively. There was also significant reverse correlation between total fatigue score with total sleep quality score (p=0.031) and with an increase in fatigue, sleep quality was reduced.

**Discussion:**

The findings of this study may be used to design interventional measures for fatigue and medical care to improve quality of sleep in pregnancy.

## INTRODUCTION

Fatigue is one of the most common and persistent complaints reported by pregnant women. According to physiological principles and theories, energy imbalance due to hormonal and metabolic changes, mental and physical adaptation to pregnancy or illness causes fatigue during pregnancy^1^. Fatigue is ‘an overwhelming, sustained sense of exhaustion, and decreased capacity for physical and mental work^2^.

More severe fatigue during pregnancy can increase the risk of preterm labor^3^, prolonged labor, operative vaginal delivery, cesarean section, and postpartum depression^4,5^. Poor sleep quality during pregnancy may increase the risk of depression in the last trimester of pregnancy^6^. Reports have also shown the association of poor sleep quality with negative pregnancy outcomes^7^.

Adverse effects of fatigue were addressed in previous studies but they were not thoroughly explored^10^. A review of medical databases did not reveal an assessment of fatigue in different trimesters of pregnancy. No accurate data on the prevalence of fatigue in different trimesters of pregnancy and its relationship with sleep quality was also found in Iranian database. Ensuing that fatigue is a common problem during pregnancy^8^ and is related to sleep quality^9^ can help to plan effective interventions to reduce fatigue and subsequently improve sleep quality. The hypothesis of this study is that fatigue will have a significant relationship with sleep quality. Therefore, the present study aimed to determine the severity of fatigue in different trimesters of pregnancy and its relationship with sleep quality in pregnant women.

## MATERIAL AND METHODS

### Study design and participants

This was a cross-sectional study. The statistical population consisted of 605 pregnant women visiting the health centers of Tabriz in Iran from late January to late April 2017. The study received ethical approval (ethics code: ir.tbzmed.rec.1392.244) from Tabriz University of Medical Sciences. Inclusion criteria were singleton pregnancy, living at the same house with the spouse at the time of the study (according to Iranian culture, the presence of pregnant woman in her parents’ house for more care leads to double emotional, psychological and physical support, and causes bias in measuring the fatigue and sleep score^11^), the possibility of contacting pregnant women via phone calls (the researcher extracted the list of pregnant women covered by the centers along with their phone numbers through the Integrated Health System (SIB) and randomly selected the women based on the sample size determined for each center), and no-night shift occupation type. Exclusion criteria were fetal malformations, history of depression (not during pregnancy), conflict in marriage, infertility history, history of referral to a physician due to psychological disorders, history of drug use or hospitalization due to medical problems during pregnancy (e.g., thyroid disease, hypertension and high risk pregnancy), history of mental illness (especially depression in the first-degree relatives), and stressors (e.g., death of loved ones and change in residence in the past six months).

### Sampling

The two-stage cluster sampling method was used to select the samples. First, one-third of health centers were randomly selected from 85 centers in the http://www.random.org website. Then, a list of eligible pregnant women and their phone numbers was prepared. The sample was selected using stratified random sampling method. The selected pregnant women were contacted. Objectives of the research were explained to them. The women who were willing to participate in the study were asked to attend the health center at a certain time. Demographic data as well as inclusion and exclusion criteria were assessed. If they were eligible for the study, objectives of the study, advantages, results, data confidentiality, and the research method were explained to them in details. Informed consent forms were collected, so that they were allowed to participate in the study.

### Data collection instruments

202 women in the first trimester, 198 in the second trimester and 205 in the third trimester completed demographic questionnaire, Pittsburgh sleep quality index (PSQI), and multidimensional assessment of fatigue (MAF).

MAF is a self-report scale consisting of 16 items and measures fatigue in 4 subscales. Questions 1 and 2 measure degree and severity of fatigue, question 3 assesses fatigue-induced distress, questions 4-14 evaluate effect of fatigue on daily activities (e.g., housework, cooking, bathing, job, social activity, sexual activity, leisure time, shopping, walking exercise, and other exercises), and questions 15 and 16 measure time of fatigue. The responses delineate a model of fatigue in the past week. Finally, global index of fatigue (GFI) is calculated^12^. This instrument was also used in the study by Najafi et al. (2010)^13^ who reported acceptable validity and reliability of the instrument.

PSQI assesses attitude toward sleep quality over the past 4 weeks. Seven subscales are measured in this index, namely subjective sleep quality, sleep latency, sleep duration, habitual sleep efficiency, sleep disturbance, use of sleeping medication, and daytime dysfunction. Range of scores is between 0 and 3. Total score is calculated by sum of mean scores of these seven subscales ranging from 0 to 21. The higher the score, the poorer the quality of sleep. Scores above 5 indicate poor sleep quality. Reliability of the scale was calculated as 0.83. Designers of the scale reported acceptable validity in patients vs. control group (sensitivity=89.6% and specificity=86.5%)^14^.

### Sample size

Sample size was calculated as 85 using the G-power based on the results of the study by Ziaei et al. (2013)^15^ considering α=0.5 and power=95%, and the correlation coefficient=0.12 of subscales of sleep disorders and sleep quality with fatigue. The sample size was later determined as 170 using cluster sampling method and design effect=2. Considering loss, 202 patients in the first trimester, 198 patients in the second trimester, and 205 patients in the third trimester of pregnancy were analyzed.

### Data analysis

SPSS was used for data analysis. Descriptive statistics (e.g., frequency, percentage, mean and standard deviation) were used to describe socio-demographic characteristics, sleep quality score and its subscales, and fatigue score. Normality of quantitative data was assessed using Skewness and Kurtosis. One-way ANOVA and Kruskal-Wallis were used to compare fatigue and its subscales between different trimesters of pregnancy. Pearson and Spearman tests were used to investigate the relationship between total fatigue score and sleep quality and its subscales. General linear model was used to investigate the relationship between demographic characteristics, fatigue score and total sleep quality score. The *p*-value less than 0.05 was considered significant.

## RESULTS

**Figure 1:**
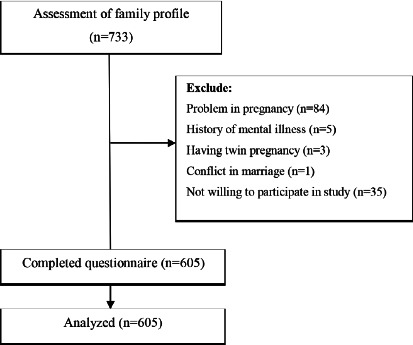
Flowchart of the study.

The number of pregnant women studied between February 2016 and May 2016 was 605 (Figure 1). Mean (SD) of age and BMI of the participant were 27.08 (5.18) and 23.89 (4.04), respectively. About half (47.4%) of the participants were undergraduates and the rest (52.6%) had either a diploma or a university degree. Most participants were housewives (90%). About half of the spouses (47.3%) had a bachelor degree and the rest (52.7%) had either a diploma or a university degree. About one-third (30.4%) of the participants possessed a private house and more than a third (39%) had rented houses. About two-thirds (60.3%) of the participants reported fairy sufficient income. About half of the fetuses (45%) were male (based on sonography) and most of mothers (90.6%) and fathers (88.9%) were interested in fetal sex (Table 1).

**Table 1:** Participants’ socio-demographic characteristics (n=605).

Characteristics	Number (%) *	Characteristics	Number (%) *	Characteristics	Number (%) *
Age (years) Mean (SD)**	27.08 (5.18)	Body mass index (kg/m2) Mean (SD) **	23.89 (4.04)	Residence	
Gravid		Husband’s educational level		Personal	184 (30.4)
1	304 (50.2)	Illiterate	---	Rental	236 (39)
2	165 (27.3)	Elementary	86 (14.2)	Others****	185 (30.6)
3≤	136 (22.5)	Secondary	144 (23.8)	Sufficiency of income	
Educational level		High school	56 (9.3)	Completely sufficient	125 (20.7)
Illiterate	4 (0.7)	Diploma	163 (26.9)	Fairy sufficient	365 (60.3)
Elementary	92 (15.2)	University	156 (25.8)	Insufficient	115 (19)
Secondary	104 (17.2)	Husband’s job		Fetal sexuality	
High school	87 (14.4)	Clerk	125 (62.1)	Female	219 (36.2)
Diploma	157 (26)	Worker	170 (84.3)	Male	272 (45)
University	161 (26.6)	Shopkeeper	72 (35.6)	Unknown	114 (18.8)
Job		Others***	233 (55.7)	Husband interest in fetal sex	538 (88.9)
Housewife	547 (90.4)	Abortion history	112 (18.5)	Woman interest in fetal sex	548 (90.6)
Employed	58 (9.6)				

* Valid percent has been reported in all the variables because of missed data;

** All data indicate number (percent), unless specified;

*** Others includes occupations such as construction, painter, agriculture, etc.;

**** Others indicate residence in parents’ house, relatives’ house and corporate house.

In this study, most of pregnant women (94.2%) suffered from fatigue. Mean (SD) of fatigue score in the first, second and third trimesters of pregnancy were 23.53 (8.05), 25.78 (6.56) and 26.46 (6.72), respectively (the range of fatigue score was 1-50). Mean (SD) of fatigue severity were 9.43 (4.90), 10.77 (4.42) and 10.71 (4.63), respectively. Mean (SD) of fatigue-induced distress scores were 4.44 (2.96), 4.89 (2.61) and 5.22 (3.00), respectively. Mean (SD) of fatigue effect on daily activities were 35.15 (18.22), 37.24 (18.56), and 38.40 (21.23), respectively. Mean (SD) of fatigue duration were 4.68 (1.52), 4.65 (1.99) and 4.65 (1.99), respectively. One-way ANOVA and Kruskal-Wallis test showed a significant difference between total fatigue score and subscales of fatigue severity and fatigue distress in three different trimesters of pregnancy (*p*<0.05) (Table 2).

**Table 2:** Fatigue level and its subscales in pregnant women referring to Tabriz health centers in different trimesters of pregnancy (n=605).

Characteristic	First trimester (n=202)		Second trimester (n=198)		Third trimester (n=205)		p
	Mean(SD)*	Md(P25%-P75%) **	Mean(SD)*	Md(P25%-P75%) **	Mean(SD)*	Md(P25%-P75%) **	
Total score of fatigue (1-50)	23.53(8.05)	24(17.3-29.8)	25.78(6.56)	24.9(20.3-30.8)	26.46(6.72)	26(21.1-31.5)	<0.001^†^
Fatigue severity	9.43(4.90)	10(5-13)	10.77(4.42)	11(7-14)	10.71(4.63)	10(7-14)	0.005^†^
Fatigue-induced distress	4.44(2.96)	4.5(2-7)	4.89(2.61)	5(3-7)	5.22(3)	5(3-8)	0.032^‡^
Fatigue interference in daily activities	35.13(18.22)	31(22-48.2)	37.24(18.56)	36(23-51)	38.40(21.3)	36(22-53.7)	0.227^†^
Duration of fatigue	4.68(1.52)	5(4-6)	4.55(1.68)	5(4-6)	4.65(1.99)	5(4-6)	0.756^†^

* Mean (Standard deviation);

** Median (25 and 75 percentile);

† One-way ANOVA;

‡ Kruskal-Wallis test.

**Figure 2:**
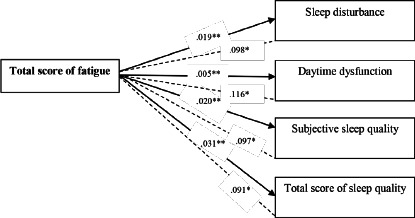
Conceptual diagram of relationship between total fatigue score with sleep quality and its components in pregnant women referring to Tabriz health centers (n=605). *Numbers represent the correlation coefficient; **Numbers represent the p-value.

The results showed significant reverse correlation of total fatigue score with total sleep quality score and subscales (Table 3) of mental quality of sleep, sleep disturbances and daily dysfunction, so that with an increase in fatigue, sleep quality was reduced (Figure 2).

**Table 3:** Relationship between total fatigue score with sleep quality and its components in pregnant women referring to Tabriz health centers (n=605).

Variables	Mean (SD)*	Md (P25%-P75%) **	Correlation with total score of fatigue	
			r	p
Total score of sleep quality	6.56 (3.24)	6 (9– 4)	0.091	0.031^†^
Subjective sleep quality	1.23 (0.89)	1 (2-1)	0.097	0.020^‡^
Sleep latency	1.51 (0.96)	1 (2-1)	0.063	0.134^‡^
Sleep duration	0.35 (0.74)	0 (0-0)	0.014	0.743^‡^
Habitual sleep efficiency	0.52 (0.75)	0 (1-0)	-0.013	0.762^‡^
Sleep disturbance	1.49 (0.62)	1 (2-1)	0.098	0.019^†^
Use of sleeping medication	0.20 (0.13)	0 (0-0)	0.004	0.917^‡^
Daytime dysfunction	1.46 (0.84)	1 (2-1)	0.116	0.005^†^
Total score of fatigue	25.22 (7.26)	24.9 (30.5-19.7)	--	--
Fatigue severity	10.29 (4.69)	10 (14-7)	--	--
Fatigue-induced distress	4.85 (2.88)	5 (7-2)	--	--
Fatigue interference in daily activities	36.93 (19.42)	34 (51-22)	--	--
Duration of fatigue	4.63 (1.74)	5 (6-4)	--	--

The total score of sleep quality has a score ranging from zero to 21. The lower score shows the better situation of sleep. The total possible range of fatigue scores is 1 (no fatigue) to 50 (severe fatigue).

* Mean (Standard deviation);

** Median (25 and 75 percentile);

† Pearson test;

‡ Spearman test.

Unadjusted general linear model found the significant relationship between total fatigue score, fatigue severity, fatigue-inducing distress, gestational age, place of residence, gender of the fetus (based on ultrasound), and total sleep quality score during pregnancy regardless of the trimester of pregnancy. The adjusted general linear model showed a significant relationship between three variables of gestational age (third trimester), place of residence (other) and gender of fetus (female) (based on ultrasound) and total sleep quality score. So that these variables reduced the quality of sleep in pregnant women. The model predicted 25.1% of variance of sleep quality in pregnant women (Table 4).

**Table 4:** Relationship between socio-demographic characteristics and fatigue score and its subscales with total sleep quality score in pregnant women based on general linear model (n=605).

Variables	Unadjusted		Adjusted	
	β (CI95%)	*p*-value	β (CI95%)	*p*-value
Total score of fatigue	0.04 (0.004 to 0.08)	0.031	0.03 (−0.14 to 0.08)	0.564
Fatigue severity	0.06 (0.002 to 0.11)	0.044	0.01(−0.12 to 0.15)	0.829
Fatigue-induced distress	0.12 (0.03 to 0.21)	0.009	0.10 (−0.08 to 0.29)	0.265
**Gestational age** (third trimester)	--	--	--	--
First trimester	−3.4 (−3.9 to −2.8)	<0.001	−3.05 (−3.72 to −2.38)	<0.001
Second trimester	−2.8 (−3.3 to −2.2)	<0.001	−2.84 (−3.43 to −2.25)	<0.001
**Residence (**others*)	--	--	--	--
Personal	−2.8 (−5.1 to −0.6)	0.014	−4.70 (−6.85 to −2.54)	<0.001
Rental	−2.6 (−4.9 to −0.3)	0.025	−4.44 (−6.59 to −2.29)	<0.001
Mother’s parents’ home	−1.2 (−4.6 to 2.2)	0.487	−2.68 (−5.78 to 0.41)	0.089
Father’s parents’ home	−3.2 (−5.5 to −0.9)	0.005	−5.05 (−7.20 to −2.89)	<0.001
**Fetal Sexuality (**unknown**)**	--	--	--	--
Male	1.6 (0.9 to 2.3)	<0.001	0.52 (−0.21 to 1.25)	0.163
Female	2.3 (1.6 to 3)	<0.001	1.11 (0.35 to 1.87)	0.004

The total score of sleep quality has a score ranging from zero to 21. The lower score shows the better situation of sleep. The total possible range of fatigue scores is 1 (no fatigue) to 50 (severe fatigue).

* Others indicate residence in parents’ house, relatives’ house and corporate house; Adjusted R^2^= 0.251.

## DISCUSSION

The present study aimed to investigate fatigue in different trimesters of pregnancy and its relationship with sleep quality of pregnant women visiting health centers of Tabriz. The results showed that more than 90% of pregnant women in the first, second, and third trimesters of pregnancy are affected with fatigue. The results showed significant direct correlation of fatigue score with total sleep quality score and subscales of subjective sleep quality, sleep disturbance and daytime dysfunction, so that with an increase in fatigue, sleep quality was reduced.

Studies reported a significant relationship between severe fatigue and increased rate of cesarean section, preterm birth and low birth weight^16^. However, pregnancy fatigue was not examined in caring for pregnant women in health centers or even in the gynecology clinics. Therefore, effective measures and training programs were not planned to reduce pregnancy fatigue.

The results of the present study showed higher fatigue score in the third trimester of pregnancy. These results were consistent with the results of study by Rodriguez et al. (2001)^17^ who found persistent pregnancy fatigue in 90% of pregnant women. In addition to severe fatigue in the last trimester of pregnancy, high prevalence of sleep disorders was reported in the third trimester of pregnancy^8,18^.

A significant relationship was found between fatigue and total sleep quality score in the present study; with an increase in fatigue level, sleep quality was reduced. These results were consistent with the results of the study by Tsai et al. (2012)^19^ who assessed 48 nulliparous women in the third trimester of pregnancy. They showed that length of sleep in the previous night had a significant and inverse correlation with morning and afternoon fatigue based on the adjusted model. Hall et al. (2009)^20^ also found that hours of sleep per night had an inverse correlation with fatigue.

Beebe and Lee (2007)^18^ showed that sleep duration and frequency of waking up during night sleep were correlated with fatigue during pregnancy. Sleep disorder and fatigue are common complaints of women during pregnancy^21^. Sleep disturbances, sleep disorders and fatigue during pregnancy can affect quality of life, social relationship, family relations, and career of the pregnant women and even their spouses^22^.

Gestational age was a predictor of sleep disorder in the present study. This finding was consistent with the results of a meta-analysis in Canada^23^ that found a decline in sleep quality as pregnancy progressed. Pregnancy problems mostly appear in the last trimesters of pregnancy. The incidence of sleep disorders also increases in the last trimesters of pregnancy.

Female fetus is also another predictor of sleep disorder in the present study. Unfortunately, the review of literatures found no evidence on this issue. Therefore, relevant studies are discussed. The results of this study were consistent with the results of the study by Malakoti et al. (2014)^24^ who assessed the health-promoting lifestyle and its predictors in pregnant women. They also found the significant relationship of fetal gender with health-promoting lifestyle that consequently reduced stress during pregnancy. A significant relationship was found between sleep quality and stress in pregnant women^25^. Therefore, it can be assumed that lack of interest in the fetus sex may increase the perceived stress in the mother and this can cause sleep disturbance in this group of women and still a preference for son is rooted in Iranian culture^26^. Given the Iranian culture and the preference of the boy sex as a guarantor of the survival of the family^27^, this may be one of the possible reasons for this relationship in our study.

Place of residence (living in a private house, rental house, parental house) was another predictor of sleep disorder in the present study. Living in organizational houses and with other people except family members increases the risk of sleep disorders during pregnancy, which is higher than the risk of living in a private house, rental house and parental since living in parental house or a private house boosts financial confidence, which eliminates psychological burden of financial concerns that help pregnant women sleep better at nights. Unfortunately, a similar study was not found in this regard.

One of the strengths of this study was large sample size and the use of standard scientific questionnaires.

### Clinical implications

Considering the results of this study and the reverse relationship between fatigue and sleep quality in this study, supportive interventions for prevention of fatigue and improved sleep quality must be considered by midwives as healthcare providers as an important issue in pregnancy.

### Limitations

Since the participants consisted of women without medical problems, this reduces the generalizability of the findings. Given this is a cross-sectional study, the relationship shown between fatigue and sleep quality, and some socio-demographic and obstetric characteristics do not necessarily indicate a causal relationship. It is recommended that fatigue and sleep disorders be explored in pregnant women with medical problems and with longitudinal method in future studies. Since limited studies have addressed the effect of socio-demographic on sleep quality of pregnant women, it is suggested to carry out similar studies in this area.

## CONCLUSION

Pregnant mothers experience excessive fatigue in different periods of pregnancy. Pregnancy fatigue is related to sleep quality. Therefore, it is important to assess effective factors in fatigue and sleep quality. Hopefully, the results of this study highlight that the health system should plan effective measures for assessment of fatigue and sleep disorders in pregnant women.
